# N^6^-methyladenosine levels in peripheral blood RNA: a potential diagnostic biomarker for colorectal cancer

**DOI:** 10.1186/s12935-024-03289-2

**Published:** 2024-03-05

**Authors:** Chunying Zhang, Jiadi Chen, Jingyi Ren, Xiaoyu Li, Yaqin Zhang, Bihan Huang, Yihan Xu, Luyan Dong, Yingping Cao

**Affiliations:** Department of Clinical Laboratory, Fujian Medical University Union Hospital, Fuzhou, China

**Keywords:** Peripheral blood, N^6^-methyladenosine, Biomarker, Colorectal cancer

## Abstract

**Background:**

N^6^-methyladenosine (m^6^A) is dysregulated in various cancers, including colorectal cancer (CRC). Herein, we assess the diagnostic potential of peripheral blood (PB) m^6^A levels in CRC.

**Methods:**

We collected PB from healthy controls (HCs) and patients with CRC, analyzed PB RNA m^6^A levels and the expression of m^6^A-related demethylase genes *FTO* and *ALKBH5*, cocultured CRC cells with PB mononuclear cells (PBMCs), and constructed an MC38 cancer model.

**Results:**

PB RNA m^6^A levels were higher in the CRC than that in HCs. The area under the curve (AUC) of m^6^A levels (0.886) in the CRC was significantly larger compared with carbohydrate antigen 199 (CA199; 0.666) and carcinoembryonic antigen (CEA; 0.834). The combination of CEA and CA199 with PB RNA m^6^A led to an increase in the AUC (0.935). Compared with HCs, the expression of *FTO* and *ALKBH5* was decreased in the CRC. After coculturing with CRC cells, the PBMCs RNA m^6^A were significantly increased, whereas the expression of *FTO* and *ALKBH5* decreased. Furthermore, m^6^A RNA levels in the PB of MC38 cancer models were upregulated, whereas the expression of *FTO* and *ALKBH5* decreased.

**Conclusions:**

PB RNA m^6^A levels are a potential diagnostic biomarker for patients with CRC.

**Supplementary Information:**

The online version contains supplementary material available at 10.1186/s12935-024-03289-2.

## Background

In the world, colorectal cancer (CRC) ranks third in terms of prevalence and second in terms of mortality [1, 2]. The incidence of CRC is increasing annually, owing to recent changes in lifestyle and dietary structures [3]. Early detection, timely diagnosis, and radical surgery are crucial for the successful treatment of CRC. However, early-stage clinical symptoms are not often apparent. Most CRC patients are diagnosed at an advanced stage where surgery is not an option. Presently, the gold standard screening methods for CRC are colonoscopy and tissue biopsy [4]; however, these methods are not practical for large-scale screening for CRC owing to their high cost and poor patient compliance [5]. Moreover, CRC is commonly detected using blood-based biomarkers, such as carcinoembryonic antigen (CEA), carbohydrate antigen 125 (CA125), and carbohydrate antigen 19-9 (CA19-9) [6, 7]. However, the diagnostic performance of these biomarkers is unsatisfactory owing to low diagnostic sensitivity or specificity [8]. As a result, a blood biomarker for the clinical diagnosis of CRC is urgently needed.

N^6^-methyladenosine (m^6^A) is the most prevalent and conserved transcriptional modification [9, 10]. A methyltransferase complex composed of METTL3, METTL14, and WTAP catalyzes this modification. Additionally, FTO and ALKBH5 are m^6^A demethylases that regulate the reversibility of the m^6^A modification. Dysregulated m^6^A-related genes have been reported in CRC cells [11]. Low *FTO* expression in patient-derived CRC cell lines increases m^6^A levels in mRNAs, resulting in enhanced tumorigenicity and chemoresistance in vivo [12]. ALKBH5 plays an antitumor role in CRC by increasing the stability of FOXO3 by attenuating the level of its m^6^A modification, and FOXO3 targets miR-21 and promotes the expression of SPRY2, providing a new direction for CRC therapy [13]. Moreover, the levels of m^6^A and METTL3 were increased in CRC tissues, and high m^6^A or METTL3 levels predict poor prognosis [14]. Additionally, CRC tumorigenesis can be facilitated by the activation of the glycolysis pathway by m^6^A methylation [15]. Thus, the modification of m^6^A is crucial in the development and progression of CRC.

Patients with cancer, including gastric cancer [16], breast cancer [17], and non-small-cell lung carcinoma [18], have elevated levels of m^6^A in their peripheral blood (PB). The levels of m^6^A in PB correlate with tumor stage and can be used as diagnostic biomarkers. Moreover, these patients exhibit lower expression of m^6^A demethylase genes *FTO* and *ALKBH5* in PB. However, the diagnostic potential of the m^6^A modification for patients with CRC has not been investigated.

In the current study, we aimed to investigate the diagnostic potential of levels of PB RNA m^6^A levels for CRC by comparing the same in HCs and patients with CRC. In addition, we demonstrated in vitro that CRC cells could regulate the levels of m^6^A in PB mononuclear cells (PBMCs) and detected the levels of PB RNA m^6^A in a mouse model of CRC.

## Materials and methods

### Sample collection

PB samples were collected from 78 patients with CRC and 44 HCs without a history of primary or chronic diseases at the Fujian Union Hospital using EDTA tubes. All patients with CRC were diagnosed based on histopathology via endoscopic examination or biopsy, and PB samples were obtained at diagnosis before surgery or radio/chemotherapy. The demographic characteristics of patients with CRC and HCs are listed in Table 1 and Additional file 1: Table S1. Ethical approval (2021QH036) was granted by the Ethics Committee of Fujian Union Hospital.

**Table 1:** Correlation between the levels of m^6^A and clinicopathological characteristics in CRC

Characteristics	No. of patients	Peripheral blood m^6^A levels % (mean ± SD)	*P*
Age			
≤ 60	36	0.2884 ± 0.01005	0.3547
> 60	42	0.301 ± 0.008999	
Gender			
Female	32	0.3019 ± 0.01148	0.4092
Male	46	0.2905 ± 0.008122	
Clinical stage			
I	13	0.2707 ± 0.008552	0.1692
II	20	0.3164 ± 0.01441	
III	31	0.2889 ± 0.01018	
IV	12	0.2879 ± 0.01869	
T classification			
T1–T2	11	0.2773 ± 0.01684	0.3121
T3–T4	55	0.2974 ± 0.008176	
N classification			
N0	25	0.2869 ± 0.01146	0.4016
N1–N2	42	0.2997 ± 0.0095	
N classification			
N0–N1	47	0.2956 ± 0.009102	0.8516
N2	19	0.2925 ± 0.01294	
M classification			
M0	45	0.2922 ± 0.009224	0.2933
M1	20	0.2753 ± 0.01174	
CEA (ng/mL)			
< 5	50	0.2844 ± 0.008085	0.0380
≥ 5	26	0.3143 ± 0.01196	
CA19-9 (ng/mL)			
< 35	61	0.2871 ± 0.007505	0.0252
≥ 35	15	0.3254 ± 0.01456	

m^6^A: N6-methyladenosine, T: tumor: N: node, M: metastasis, CEA: carcinoembryonic antigen, CA19-9: carbohydrate antigen 19-9

After blood collection, 1 mL of EDTA + blood was treated with 3 mL erythrocyte lysate (Cat. No. R1010; Solarbio) twice to obtain leukocytes, then 1 mL of Trizol reagent (Cat. No. 15596-026; Invitrogen) was added to stabilize the RNA, and the samples were preserved at − 80 ℃ until RNA extraction.

### RNA isolation

Total RNA was extracted using the TRIzol reagent. The integrity of RNA was evaluated using agarose gel electrophoresis. RNA yield and purity were measured using the NanoDrop 1000 (Gene Company Limited, Hong Kong, China).

### Quantitative real-time polymerase chain reaction (qRT-PCR)

Quantitative RT-PCR was performed using the PerfectStart Green qPCR SuperMix (Cat. No. AQ601-04; TransGen Biotech, Beijing, China) and a 7500 Real-Time PCR System (Thermo Fisher Scientific). The cycling parameters were as follows: 94 ℃ for 30 s, followed by 40 cycles of 94 ℃ for 5 s, 60 ℃ for 15 s, and 72 ℃ for 10 s. To calculate the ΔCq values, the Cq value for the human actin beta [*ACTB*] reference gene and the mice actin beta [*Actb*] gene was subtracted from the original Cq value. Normalization of targeted gene expression in each independent sample was performed using the reference (*ACTB*) gene. The primers for the target genes are listed in Additional file 1: Table S2. The 2^−ΔΔCt^ method was used to calculate the absolute expression.

### Quantification of m^6^A in PB RNA

The EpiQuik m^6^A RNA Methylation Quantification Kit (Colorimetric; EpiGentek, Farmingdale, NY, USA) was used to measure m^6^A levels in total RNA by the manufacturer’s instructions. First, 80 µL binding solution was added to the assay well, and then 200 ng RNA. Following a 90-min incubation at 37 ℃, the plates were washed thrice, and the assay wells were sequentially treated with diluted solutions of the primary and detection antibodies and the enhancer. Subsequently, the color reaction was initiated by adding the developer and stop solutions to each well, and the absorbance was measured at 450 nm wavelength. Finally, the standard curve was generated to determine the m^6^A levels.

### Cell lines and cell experiments

The human CRC cell line (HCT116) was obtained from the Chinese Academy of Sciences (Beijing, China). SW480 and MC38 murine colon adenocarcinoma cells were kindly provided by Dr. Mi Zhang of the Basic Medicine of Fujian Medical University (Fuzhou, China). PBMCs were isolated from healthy human PB using the human PB lymphocyte separation medium. SW480 and HCT116 cells were cultured in RPMI 1640 medium (Gibco, Thermo Fisher Scientific) supplemented with 10% fetal bovine serum and 1%Pen/Strep (Thermo Fisher Scientific), and PBMCs were cultured in human PB lymphocyte particular medium in an incubator at 37 ℃ and 5% CO_2_.

Six-well Transwell chambers (0.4-µm pores, Corning Transwell; Corning Inc, Corning, NY, USA) were used for the co-culture assay. A total of 5 × 10^5^ HCT116 or SW480 cells were seeded in the lower Transwell chamber, and 1 × 10^6^ PBMCs were seeded in the upper chamber of the co-culture system for 48 h. PBMCs were collected separately for qRT-PCR and ELISA analyses.

### Animal study

C57BL/6 male mice (6–8-week-old) were purchased from the Sibeifu Animal Center (Beijing, China). All animal experiments were conducted following the protocols approved by the Fujian Medical University of Medicine Policy on the Care and Use of Laboratory Animals. MC38 colon carcinoma cells were injected in 100 µL phosphate-buffered saline (PBS) subcutaneously into the right flank of C57BL/6 mice (n = 14, male) to construct an MC38 murine colon carcinoma cancer model (MC38 cancer model). C57BL/6 mice were also injected with 100 µL of PBS into their right flank as the control group (n = 8). Mice were euthanized 15 day after cells/PBS injection or if the longest dimension of the tumor was as large as 1.5 cm within 20 day. Retroorbital blood was obtained from the mice immediately following euthanasia using EDTA tubes.

### Statistical analysis

Statistical analysis was conducted using IBM SPSS Statistics 20.0 (IBM Corp., Armonk, NY, USA) and GraphPad Prism version 7.0 (GraphPad Software, San Diego, CA, USA). Differences between groups were analyzed using an unpaired 2-tailed parametric Student’s *t-*test for normally distributed data. Otherwise, the nonparametric Mann–Whitney *U* tests were used to analyze the data. Data were analyzed using a one-way analysis of variance followed by Bonferroni-corrected posthoc tests to allow for comparison of more than two groups. A receiver operating characteristic (ROC) curve was plotted to determine the diagnostic value of the biomarker. The standard deviations are represented by error bars. Statistical significance was set at *P* < 0.05.

## Results

### Levels of m^6^A in the PB of patients with CRC and HCs

**Fig. 1:**
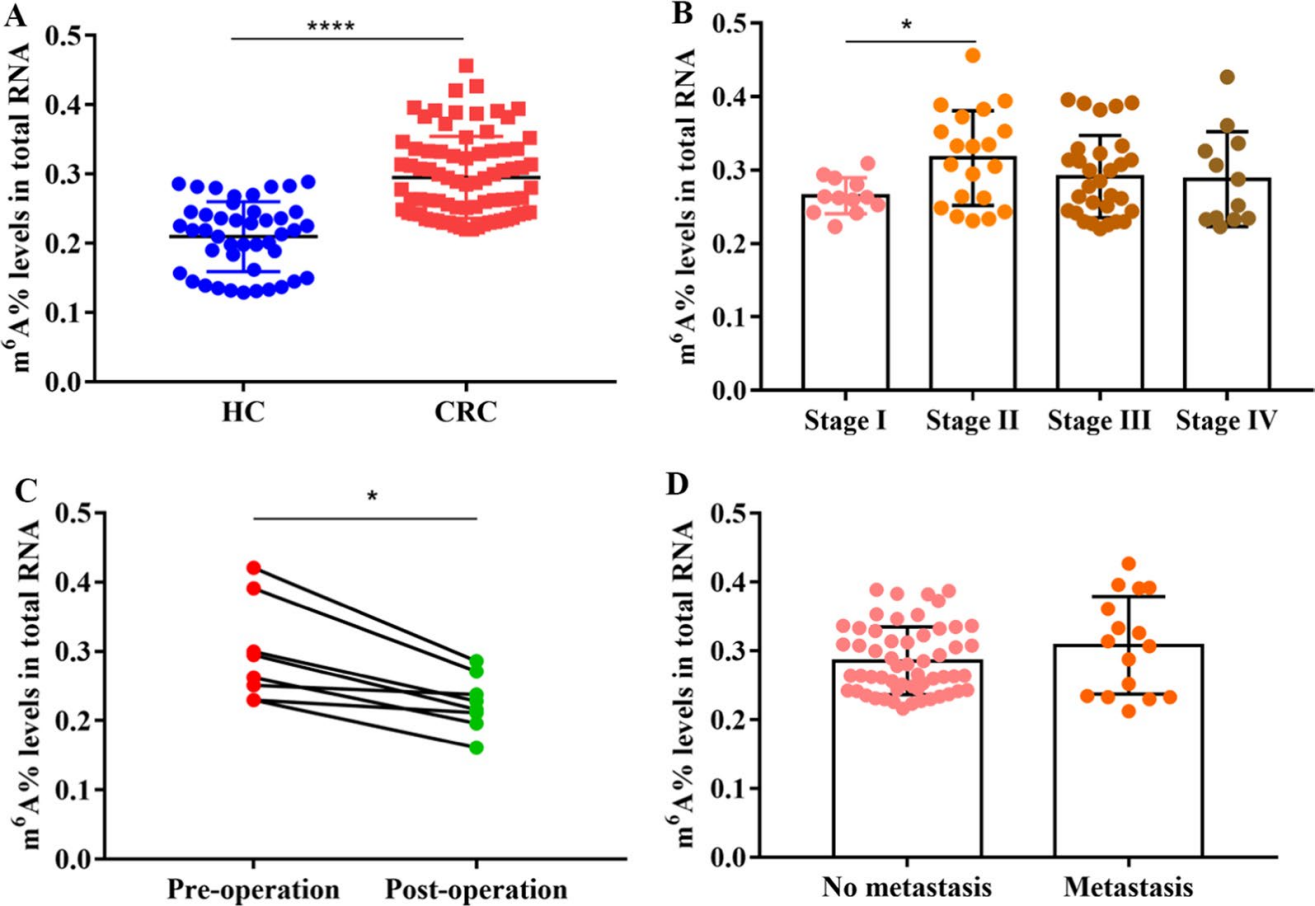
The levels of N^6^-methyladenosine (m^6^A) in RNA of the peripheral blood of healthy controls (HCs) and patients with colorectal cancer (CRC). **A** The m^6^A levels of peripheral blood RNA in HC (n = 44) and patients with CRC (n = 78); **B** m^6^A levels in peripheral blood RNA of patients with different clinical stages of CRC (stage I, n = 13; stage II, n = 20; stage III, n = 31; stage IV, n = 12); **C** the m^6^A levels of peripheral blood RNA in patients with CRC (n = 8) before surgery and after seven days of surgery; **D** comparison of m^6^A levels in peripheral blood RNA between patients with CRC with (n = 16) and without (n = 56) metastasis. The bars represent the mean ± standard deviation (SD) of the results from three replicate measurements; *P < 0.05, ****P < 0.0001. HC: healthy control, CRC: colorectal cancer

The EpiQuik m^6^A RNA Methylation Quantification Kit was used to determine the levels of PB RNA m^6^A of patients with CRC (n = 78) and HCs (n = 44). As depicted, patients with CRC had significantly higher PB RNA m^6^A levels than HCs (*P* < 0.0001) (Fig. 1A). Moreover, we investigated whether PB RNA m^6^A levels could be used to distinguish between different pathological stages of CRC (stage I, n = 13; stage II, n = 20; stage III, n = 31; stage IV, n = 12). As depicted, PB RNA m^6^A levels of the stage II group were significantly higher than those of the stage I group (*P* < 0.05) (Fig. 1B). However, PB RNA m^6^A levels of patients with CRC with or without metastasis were of no significant difference (*P* > 0.05; Fig. 1C).

Moreover, we analyzed PB RNA m^6^A levels in patients with CRC (n = 8) before and after surgery (after 7 day) to determine whether m^6^A levels could be used as a potential follow-up biomarker. The results suggested that PB RNA m^6^A levels in patients with CRC significantly decreased after surgery (*P* < 0.05; Fig. 1D). Collectively, these results indicated that PB RNA m^6^A levels in patients with CRC are upregulated with the progression of CRC and may serve as a potential follow-up biomarker after surgery for patients with CRC.

### Levels of m^6^A in PB RNA exhibit potential as a biomarker for patients with CRC

**Fig. 2:**
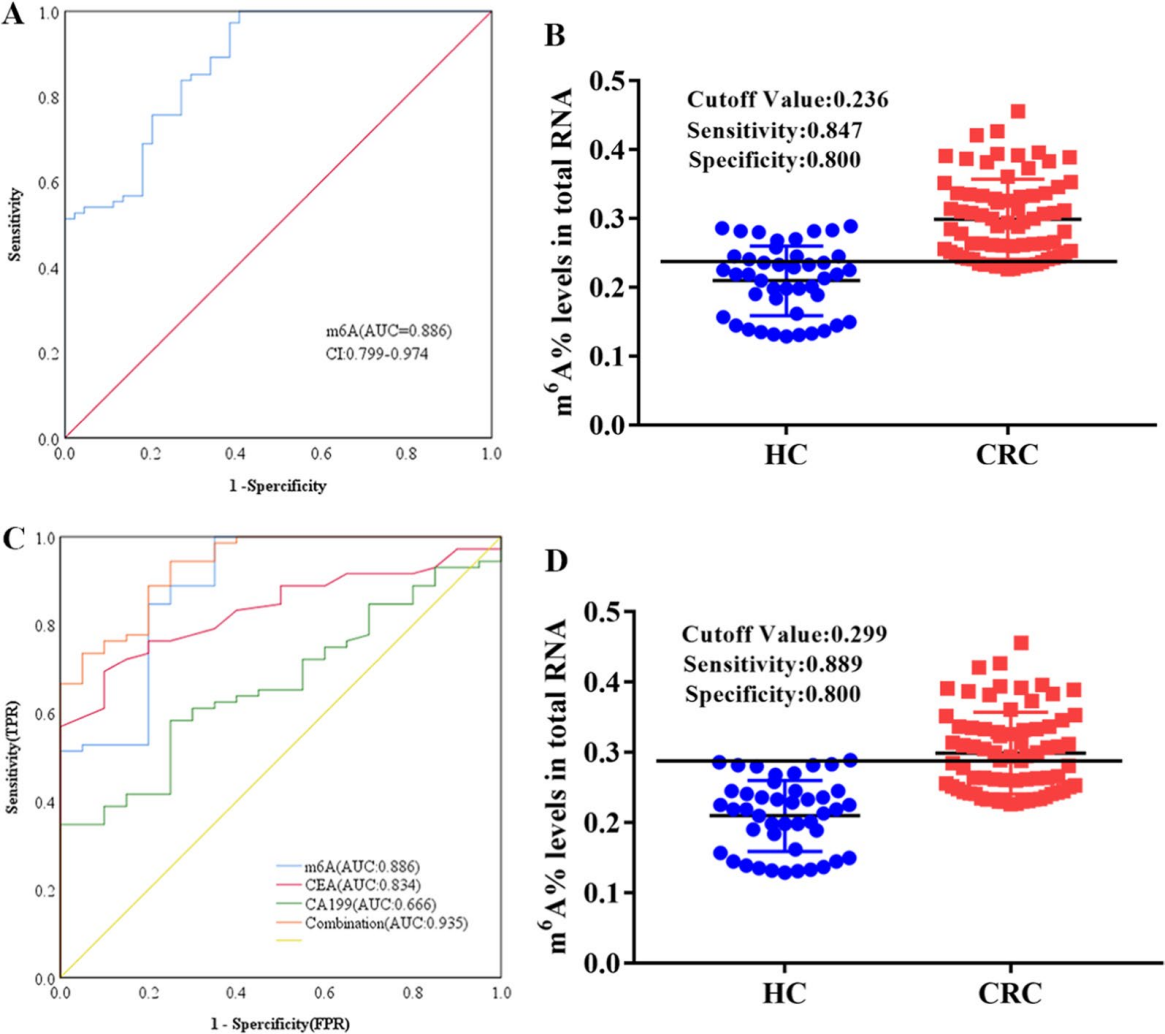
Clinical utility of m^6^A levels in peripheral blood RNA, carcinoembryonic antigen (CEA), and carbohydrate antigen 199 (CA199) in diagnosing CRC. **A** Receiver operating characteristic curve and **B** cutoff value for m^6^A for patients with CRC and HCs; **C** receiver operating characteristic curves for m^6^A, CEA, and CA199 alone or together, and **D** cutoff values for m.^6^A, CEA, and CA199 in combination for patients with CRC and HC. HC: healthy control, CRC: colorectal cancer, CEA: carcinoembryonic antigen, CA199: carbohydrate antigen 199

To determine the diagnostic value of PB RNA m^6^A levels for patients with CRC, the ROC curve was plotted. As depicted, the area under the curve (AUC) for PB RNA m^6^A levels was 0.886 (95% confidence interval CI 0.799–0.974; *P* < 0.0001) (Fig. 2A), which could be used to distinguish patients with CRC from HCs. PB RNA m^6^A had an optimal cutoff value of 0.236 (diagnostic sensitivity 84.7%; diagnostic specificity 80%) (Fig. 2B). In comparison to CEA (AUC 0.834) and CA199 (AUC 0.666), the diagnostic value of PB RNA m^6^A was significantly better (Fig. 2C). Furthermore, combining PB RNA m^6^A, CEA, and CA199 levels improved the AUC of the ROC curve to 0.935 (95% CI 0.882–0.989, *P* < 0.0001) (Fig. 2D), indicating the excellent diagnostic potential for patients with CRC.

### Expression of FTO and ALKBH5 decreased in the PB RNA of patients with CRC

**Fig. 3:**
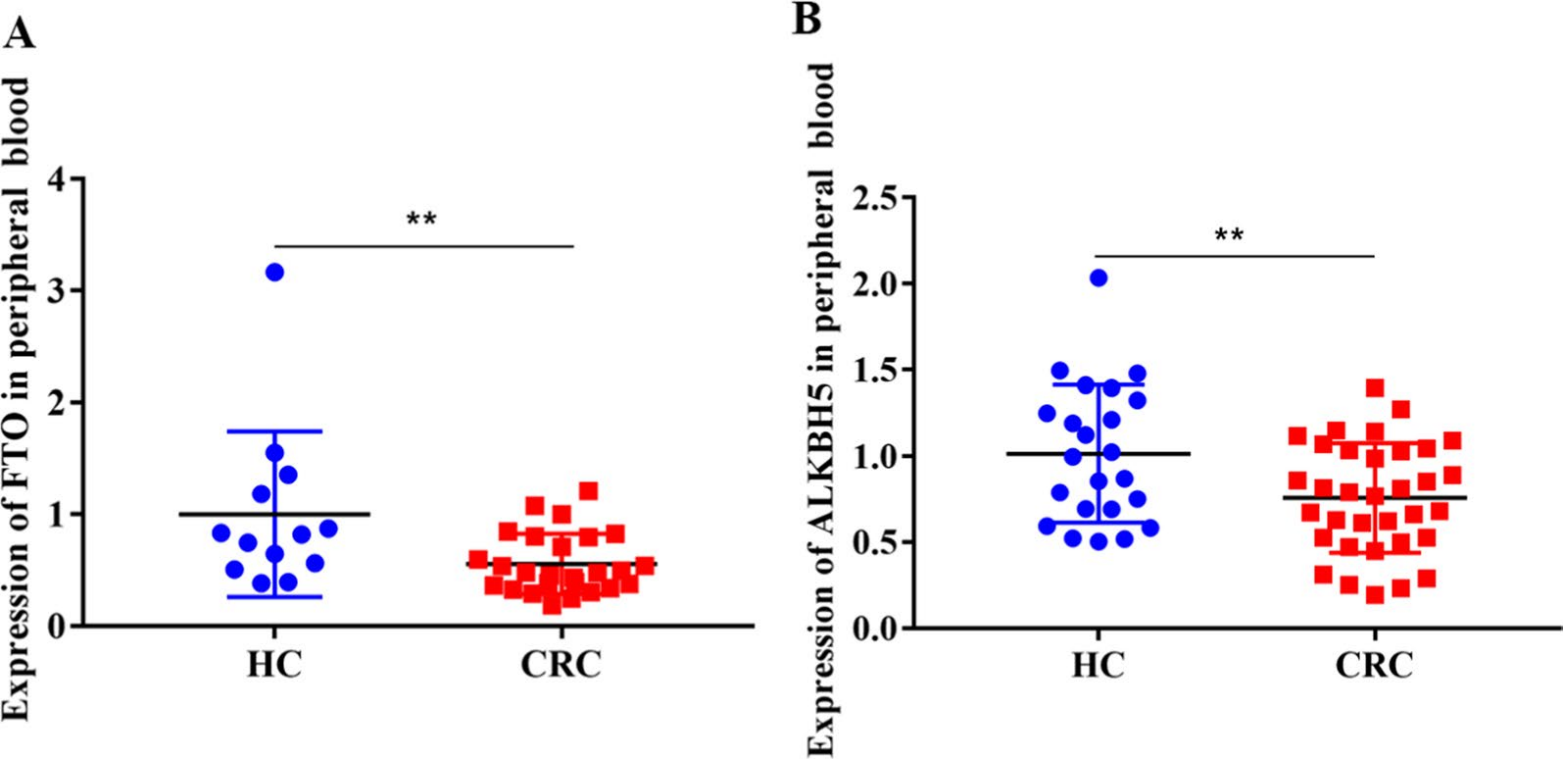
Expression levels of *FTO* and *ALKBH5* in patients with CRC. **A** Quantitative real-time polymerase chain reaction for the relative mRNA expression of *FTO* and *ALKBH5* (**B**) in the peripheral blood of patients with CRC (n = 26; n = 34) and HC (n = 13; n = 23). **P < 0.01. CRC: colorectal cancer

Next, we measured the mRNA expression of *FTO* and *ALKBH5* in the PB RNA of patients with CRC and HCs. The results suggested that both *FTO* and *ALKBH5* expression were significantly downregulated in the PB RNA of patients with CRC compared with those in HCs (*P* < 0.01) (Fig. 3A and B). These results implied that the downregulation of *FTO* and *ALKBH5* expression may be associated with elevated PB RNA m^6^A levels in patients with CRC.

### Co-culture with CRC cells increases m^6^A levels in PBMCs in vitro

**Fig. 4:**
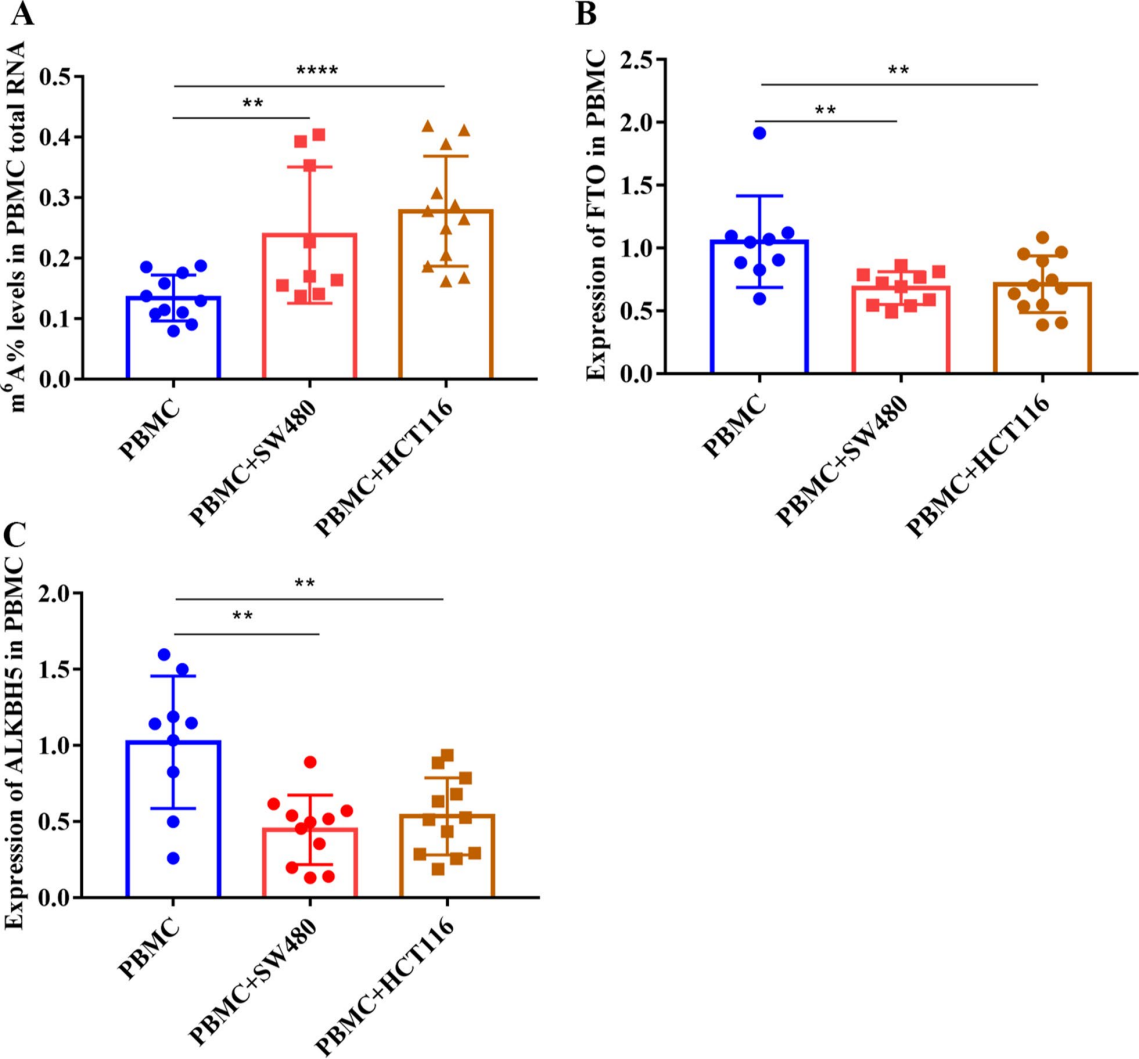
Levels of m^6^A and demethylases in blood cells cocultured with CRC cells. **A** m^6^A levels in PBMCs cocultured with or without CRC cells for 48 h; **B** mRNA levels of *FTO* in PBMCs cocultured with or without CRC cells for 48 h; **C** mRNA levels of ALKBH5 in PBMC cells cocultured with or without CRC cells for 48 h. **P < 0.01, ****P < 0.0001. CRC: colorectal cancer, PBMC: peripheral blood monocular cells

To investigate the underlying mechanisms of the upregulation of PB RNA m^6^A levels in patients with CRC, CRC cells were co-cultured with PBMCs in a Transwell chamber. The total RNA of PBMCs showed an increase in m^6^A levels upon co-culture with CRC cells, namely HCT116 and SW480 (Fig. 4A). Similarly, co-culture with CRC cells decreased mRNA expression of *FTO* and *ALKBH5* in PBMCs (Fig. 4B and C). These results indicate that the upregulation of m^6^A levels and downregulation of *FTO* and *ALKBH5* expression in PBMCs could be influenced by CRC cells.

### High m^6^A levels in the PB-RNA of an MC38 cancer model

**Fig. 5:**
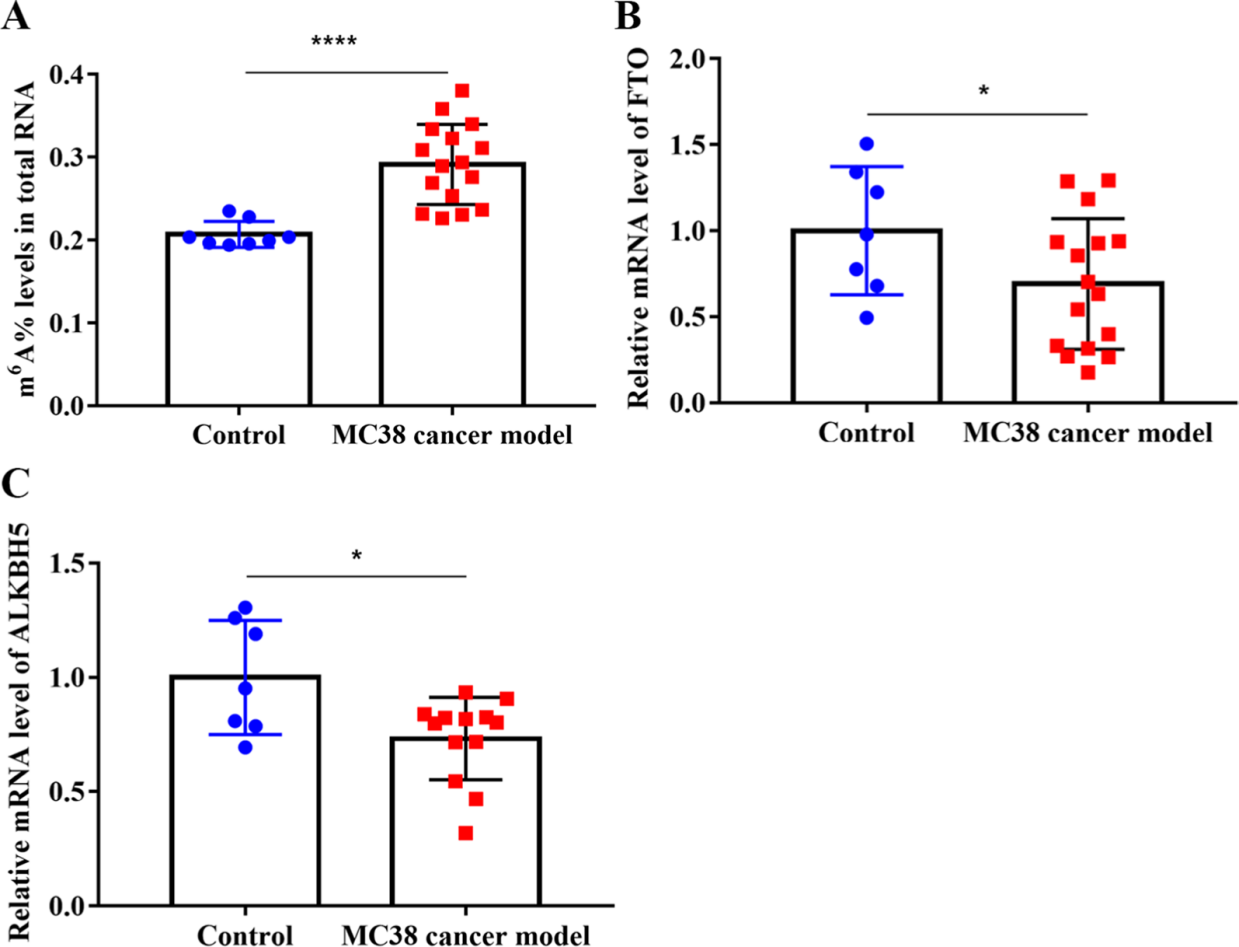
Levels of m^6^A and demethylases in the peripheral blood RNA of C57 mice. **A** Levels of m^6^A in the total peripheral blood RNA of C57 mice, control (n = 8), and MC38 cancer model (n = 14); **B**, **C** relative mRNA levels of *FTO* (B) and *ALKBH5* C in the blood of C57 mice. *P < 0.05, ****P < 0.0001. CRC: colorectal cancer

To assess whether CRC cells could increase m^6^A levels in PB-RNAs in vivo, we constructed an MC38 CRC model and measured PB RNA m^6^A levels. A significant increase in PB-RNAs m^6^A levels was observed in the MC38 cancer group when compared to the control group (*P* < 0.0001) (Fig. 5A). Moreover, the mRNA expression of *FTO* and *ALKBH5* in the PB of MC38 cancer model mice was also decreased compared with those in the PB of the control mice (*P* < 0.05) (Fig. 5B and C). These results indicate that the MC38 cancer model exhibited increased PB RNA m^6^A levels, which was also accompanied by the downregulation of *FTO* and *ALKBH5* expression in vivo*.*

## Discussion

Early diagnosis is crucial for improving CRC prognosis. Current invasive or noninvasive screening methods fail to achieve satisfactory early screening results owing to their limitations. Although multiple randomized controlled trials have shown that guaiac-based fecal occult blood testing and sigmoidoscopy reduce CRC mortality, their effectiveness in reducing CRC mortality may be diminished if patients do not repeat screening [19–22]. The serological markers CEA and CA199, currently used to diagnose CRC, have low specificity and sensitivity, both individually and in combination [23, 24]. Therefore, identifying simple and novel early diagnostic biomarkers for CRC is urgently required. In the current study, we observed that PB RNA m^6^A levels could effectively differentiate patients with CRC from HCs. The diagnostic value of PB RNA m^6^A levels was significantly improved when combined with the biomarkers CEA or CA199.

To date, few studies have investigated the diagnostic value and underlying mechanism of PB RNA m^6^A levels in patients with CRC. Our study observed that PB RNA m^6^A levels in patients with CRC were significantly increased compared with those in HCs, which is consistent with the findings of Xie et al. [25]. However, our results showed no significant differences in PB RNA m^6^A levels in patients with CRC with or without distant metastasis. The growth and progression of cancer are regulated by crosstalk between writers, readers, and erasers of m^6^A [26]. Although m^6^A levels may vary in different tumors, previous reports have suggested that PB RNA m^6^A levels in patients with gastric cancer [16], non-small cell lung carcinoma [17], breast cancer [18], and rheumatoid arthritis [27] were much higher than those in the corresponding HCs. Therefore, further investigating the diagnostic value of PB RNA m^6^A levels in other tumors and diseases is crucial.

Our results indicated that PB RNA m^6^A levels had a promising diagnostic potential for CRC. The AUC of m^6^A (0.886) for discriminating patients with CRC from HCs was the highest, followed by those of CEA (0.825) and CA199 (0.671). Moreover, CEA and CA199 in combination with PB RNA m^6^A improved the AUC to 0.935 in patients with CRC. Previous studies and meta-analyses have demonstrated that some miRNAs and circRNAs could serve as potential biomarkers for CRC; however, the AUC of the RNAs was not considered high. For example, a meta-analysis including six studies revealed that the AUC of miR-92a in the diagnosis of CRC was 0.772 [28]. Another meta-analysis, including 18 studies involving 2021 individuals, reported that the AUC of circRNA in the diagnosis of CRC is 0.81 [29]. In addition, our results showed that PB RNA m^6^A levels in postoperative CRC patients were decreased following surgery, indicating its potential as a biomarker for postoperative monitoring. However, further studies using more clinical samples are required to confirm whether it can be used as a potential follow-up biomarker. Overall, our results indicate that PB RNA m^6^A levels can act as a better diagnostic biomarker for CRC than the currently used biomarkers.

In the current study, we observed that the expression of both *FTO* and *ALKBH5* was decreased in patients with CRC compared with that in HCs. There may be a relationship between the increased PB RNA m^6^A levels in patients with CRC and the downregulation of *FTO* and *ALKBH5*. Moreover, co-culture with CRC cells results in an increase in m^6^A levels and a decrease in *FTO* and *ALKBH5* expression in PBMCs. A previous study has reported that imbalanced regulation of m^6^A strongly confers immune destruction and tumor evasion [30]. Consistently, in our study, PB RNA m^6^A levels in the MC38 cancer model were significantly increased and were accompanied by a decrease in the expression of *FTO* and *ALKBH5*. These results indicate that increased PB RNA m^6^A levels induced by CRC may be owed to the decreased expression of *FTO* and *ALKBH5.*

Our study has some limitations as no significant correlation was observed between PB RNA m^6^A levels and expressions of *FTO* or *ALKBH5* in the total RNA of PB cells. The possible reasons for the lack of correlation could be: (1) other unidentified methylases and demethylases that require further exploration [31] are also involved in the regulation of m^6^A; (2) the regulation of m^6^A in the total RNA of PB cells may require the participation of methyltransferases; (3) interactions between methyltransferases and demethylases and their regulatory factors may also contribute to the changes in m^6^A level. Therefore, changes in PB RNA m^6^A levels may not solely depend on the expression of the demethylases investigated in this study. Further investigation is needed to understand the mechanism behind the upregulation of m^6^A levels and the downregulation of demethylases induced by tumors.

## Conclusions

In conclusion, our results revealed that PB RNA m^6^A levels in patients and the mouse model of CRC are accompanied by downregulation of the demethylases *FTO* and *ALKBH5* at the transcriptional level. Our research suggests that PB RNA m^6^A levels are a promising biomarker for CRC diagnosis.

### Supplementary Information

**Additional file1: Table S1.** Clinicopathological characteristics in HC. **Table S2.** Quantitative real time PCR primers used in this study.

## Data Availability

The datasets used and analyzed during the current study are available from the corresponding author on reasonable request.

## Abbreviations

AUC: The area under the curve
CA125: Carbohydrate antigen 125
CA19-9: Carbohydrate antigen 19-9
CEA: Carcinoembryonic antigen
CRC: Colorectal cancer
HC: Healthy control
m^6^A: N^6^-methyladenosine
PB: Peripheral blood
PBMC: Peripheral blood mononuclear cell
PBS: Phosphate-buffered saline
ROC: Receiver operating characteristic
