# Correction to: N6‑methyladenosine levels in peripheral blood RNA: a potential diagnostic biomarker for colorectal cancer

**DOI:** 10.1186/s12935-024-03303-7

**Published:** 2024-04-05

**Authors:** Chunying Zhang, Jiadi Chen, Jingyi Ren, Xiaoyu Li, Yaqin Zhang, Bihan Huang, Yihan Xu, Luyan Dong, Yingping Cao

**Affiliations:** Department of Clinical Laboratory, Fujian Medical University Union Hospital, Fuzhou, China

**Correction to: Cancer Cell International (2024) 24:96**

10.1186/s12935-024-03289-2

In this article [1], the order of the corresponding author appeared in the author list was incorrect. Prof. Yingping Cao should be listed as the last author. Chunying Zhang and Jiadi Chen were equally contributed to this work. Thus, they should be listed as the co first authors.

The original article has been corrected.
