# Supplementary material for: N6-methyladenosine levels in peripheral blood RNA: a potential diagnostic biomarker for colorectal cancer
Source: Cancer Cell Int. 2024 Mar 5;24:96. doi: 10.1186/s12935-024-03289-2 (PMC10913687; doi:10.1186/s12935-024-03289-2)
Supplement: Supplementary file 1 — Additional file1: Table S1. Clinicopathological characteristics in HC. Table S2. Quantitative real time PCR primers used in this study. [file 12935_2024_3289_MOESM1_ESM.docx]

Supplementary Table 1. Clinicopathological characteristics in HC

| **Characteristics** | **No. of HC** | **Peripheral blood m^6^A levels % (mean ± SD)** | ***P*** |
| --- | --- | --- | --- |
| Age |  |  |  |
| ≤60 | 29 | 0.2087 ± 0.01028 | 0.8636 |
| >60 | 15 | 0.2115 ± 0.01064 |  |
| Gender |  |  |  |
| Female | 19 | 0.2077 ± 0.01343 | 0.8308 |
| Male | 25 | 0.2111 ± 0.008929 |  |

Abbreviations: HC, healthy control.

Supplementary Table 2. Quantitative real-time PCR primers used in this study

| **Targets** |  | **Nucleotide sequence (5'->3')** |
| --- | --- | --- |
| Human *ACTB* | Forward | TGACGTGGACATCCGCAAAG |
|  | Reverse | CTGGAAGGTGGACAGCGAGG |
| Human *FTO* | Forward | CTGGAAGCACTGTGGAAGAAG |
|  | Reverse | GCAAGGATGGCAGTCAAGATT |
| Human *ALKBH5* | Forward | CTCTTCAGCCAGGACGAGTG |
|  | Reverse | CCGTAAGTGTAGCCTTCGCC |
| Mouse *Actb* | Forward | TGTCCACCTTCCAGCAGATGT |
|  | Reverse | AGCTCAGTAACAGTCCGCCTAG |
| Mouse *FTO* | Forward | GGACATCGAGACACCAGGATT |
|  | Reverse | GGAACTAAACCGAGGCTGTGA |
| Mouse *ALKBH5* | Forward | AACCTGTGCTTTCTCTGCCG |
|  | Reverse | TCGCGGTGCATCTAATCTTGT |
